# Approaching human visual perception through AI-based representation of figure-ground segregation

**DOI:** 10.3389/fpsyg.2026.1768533

**Published:** 2026-02-27

**Authors:** Chakkai Yip, Ezekiel Moroze, Shigeaki Nishina, Arash Yazdanbakhsh

**Affiliations:** 1 Computational Neuroscience and Vision Laboratory, Department of Psychological and Brain Sciences, Boston University, Boston, MA, United States; 2 Honda Research Institute Japan Co., Ltd., Wako-shi, Saitama, Japan; 3 Graduate Program for Neuroscience, Boston University, Boston, MA, United States; 4 Center for Systems Neuroscience, Boston University, Boston, MA, United States

**Keywords:** AI saliency mapping, border-ownership, contour junctions, convolutional neural networks, feedforward processes, figure-ground segregation, partial occlusion

## Abstract

**Introduction:**

Understanding how the visual system assigns borders to foreground objects is central to figure–ground perception, yet the computational principles underlying this process are still under investigation.

**Methods:**

We trained multiple convolutional neural network (CNN) architectures on simple overlapping/occlusion stimuli and tested them on systematically degraded contours to probe how border-ownership (BOS) inference depends on available border context.

**Results:**

Across networks, BOS could be inferred from feedforward computations even under degraded conditions, but performance showed a strong dependence on junction-like configurations, indicating that geometric context contributes more than isolated edges. Accuracy increased approximately linearly with the amount of contextual information provided by fragmented borders, and representation analyses revealed a hierarchical progression from local edge responses to more spatially coherent, BOS-specific features.

**Discussion:**

Together, these results delineate which aspects of BOS can emerge from hierarchical feedforward processing and suggest that additional mechanisms such as horizontal and feedback interactions may reduce the visual information required for robust figure-ground segregation.

## 1 Introduction

Border-ownership (BOS) refers to the process by which the brain determines which side of a border belongs to an object in a figure, which plays a key role in figure-ground segregation. Studies have identified BOS-selective neural activity in primate early visual areas for both artificial and natural stimuli (Zhou et al., 2000; Hesse and Tsao, 2016), yet full understanding of cue integration and representation in biological visual networks is limited. Since convolutional neural networks (CNNs) share several key properties with the visual pathway, we can use them as a framework to explore how BOS representations might arise through hierarchical processing and to provide perspectives on the mechanisms that support figure–ground segregation in the brain.

Analogous to how receptive field size grows in higher visual areas, CNNs analyze larger patches of an image through their processing hierarchy due to increasing spatial integration across layers. Each stacked bundle of convolution-nonlinearity-pooling can be considered analogous to a single visual area forming a processing hierarchy (Lindsay, 2021). Early layers function similarly to simple cells by responding to simple stimuli like edge and orientation, while processed features become more complex deeper in the CNN in a manner analogous to visual processing. As such, the activation in deeper CNN layers could resemble the recruitment of higher visual areas as measured by fMRI (Güçlü and Van Gerven, 2015). CNNs utilize algorithms in part similar to feedback processing in the visual system for learning during the training process but operate exclusively using feedforward processing after training. Comparatively in the visual system, feedback connections between areas with different receptive fields such as V1, V2, and V4 play a critical role during the representation of scenes (Yazdanbakhsh and Gori, 2008, 2011; Sherbakov and Yazdanbakhsh, 2013). The fixed operation of CNNs after training makes it impossible for them to use feedback signaling to process new data. Nevertheless, studies have indicated strong correlation between activation of deeper layers in CNNs and spiking activity of real neurons in higher visual areas during object recognition tasks (Yamins et al., 2014; Cadena et al., 2019) and naturally emerging BOS signals during video prediction (Ye et al., 2025). Therefore, despite having a few differences, we can exploit the similarities between CNNs and the visual system to offer insight into the representations of BOS in the visual system.

Beyond simple one-to-one feedforward architectures, other modifications have been applied to achieve better performance in CNNs. Residual learning is a common technique that was developed to address degradation problems in neural networks through skip connections that maintain identity mappings in early layers (He et al., 2016). From a neuronal perspective, the functional role of these shortcut pathways can be viewed as analogous to forms of feedforward cortical modulation. In primate vision, V1 sends direct projections to V4 through pathways that bypass V2 (Nakamura et al., 1993). Similarly, pyramidal cells from neocortex layer V often bypass the intermediate layer and project long range axons to subcortical areas (Douglas and Martin, 2004), which resemble the identity-preserving characteristics of residual CNN architectures. Theory in predictive coding also suggests that cortical circuits send predictions from top to bottom while sending error signals back (Friston, 2005).

Multiscale integration has also been found to be a significant property in terms of BOS, where neurons with varying connectivity operate at multiple scales to form a complete representation. In V1, the receptive field center is defined by feedforward signals with laterally mediated near-surround and feedback-shaped far-surround areas (Angelucci and Bressloff, 2006). Computational models formalized BOS through this receptive field paradigm using large field grouping (G) units that pool over extended regions and send feedback to small-field border units, creating size-tolerant, context-dependent BOS (Craft et al., 2007). Other complementary models cast BOS as cross-scale competition, where units that share the same retinotopic center, but different spatial scales compete through mutual suppression, allowing the scale that best explains the configuration to dominate (Layton et al., 2012, 2014).

In this work, we exploited the similarities between CNNs and primate visual systems to interrogate similar mechanisms underpinning BOS representation. By investigating mechanistic differences between feedforward CNNs and visual systems, we provided insight into the specific roles of horizontal and feedback connections in BOS representation. Ultimately, we aim to provide a computational lens for interpreting BOS phenomena and help clarify which aspects of visual organization can emerge from hierarchical processing.

## 2 Methods

To investigate whether CNNs can learn BOS cues from controlled geometric configurations, we developed sets of synthetic stimuli containing overlapping shapes with occlusion relationships. In this work, we use the rectangle as our base stimulus class since it has been widely employed in psychophysical and electrophysiological studies, both in the domains of BOS and in transparency perception (Watanabe and Cavanagh, 1993a,b; Zhou et al., 2000; Zhang and Von Der Heydt, 2010). The rectangle stimulus pattern provides a relatively minimal and well-controlled geometry, which leads to a reliable generation of unambiguous T-junctions and occlusion relationships. By employing this well-defined stimulus class, we can compare our findings to prior biological studies while also minimizing possible confounds introduced by more complicated or naturalistic shape statistics.

### 2.1 Stimuli generation

**Figure 1:**
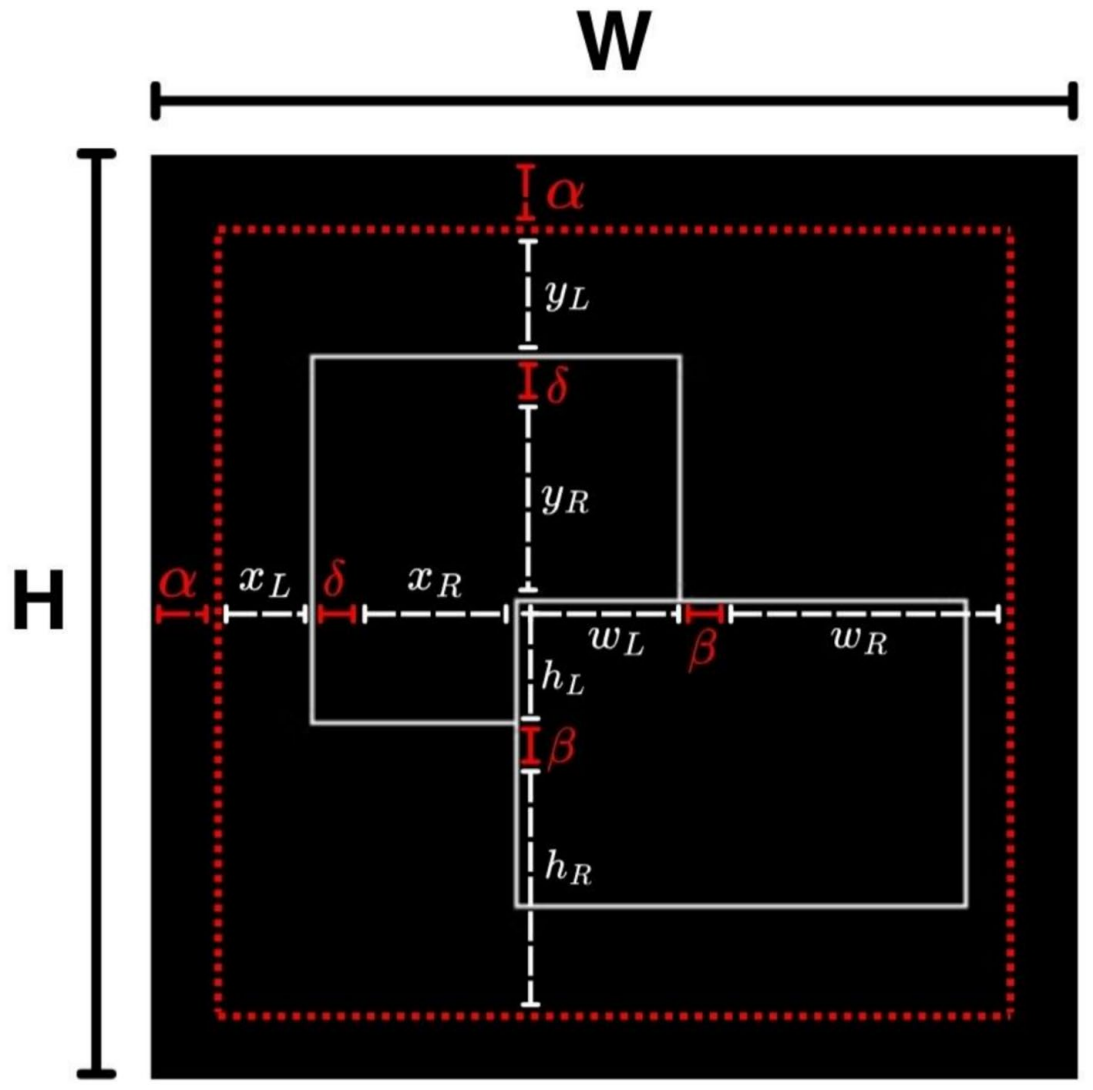
Geometric parametrization of stimulus generation. The black frame shows the image size H×W . The red dash line marks a fixed margin where no object pixels are generated and white dash line represents randomly sampled variables drawn from uniform distribution. The parameters $x_{L},y_{L},x_{R},y_{R},w_{L},h_{L},w_{R},h_{R}$ control the size and position of the two rectangles, while α,β,δ specify the fixed displacement constraints. All parameters are sampled within the ranges specified in the Methods.

In order to produce images with interpretable BOS properties to the network, we systematically generated datasets of two overlapping rectangles with randomized size, relative position, and degree of overlap to represent the naturalistic variability in occlusion relationships. For each image in the dataset with fixed resolution ( H=W=227 ) according to the input requirements of the network, we defined the left rectangle parameters ( $x_{L},y_{L},w_{L},h_{L}$ ) and the right rectangle parameters ( $x_{R},y_{R},w_{R},h_{R}$ ) by constrained uniform sampling. The $x_{L},y_{L},x_{R},y_{R}$ represented the starting position (top-left corner) of the rectangles, and $w_{L},h_{L},w_{R},h_{R}$ was the width and height of the rectangles. There were several other parameters involved that guaranteed in-bounds placement and proper overlap: α signified the minimum distance between the image margin and the position of the rectangle, δ represented the minimum interior offset of the right rectangle relative to the left, and β was the minimum extension beyond the overlap region so that it is not fully enclosed or masked by the left rectangle. An illustration of the geometric parameterization and the distinction between fixed displacement constraints and randomly sampled variables is shown in Figure 1. In our experiments, we set α=20 , β=3 , δ=5 , $w_{\min}=h_{\min}=10$ .

**Figure 2:**
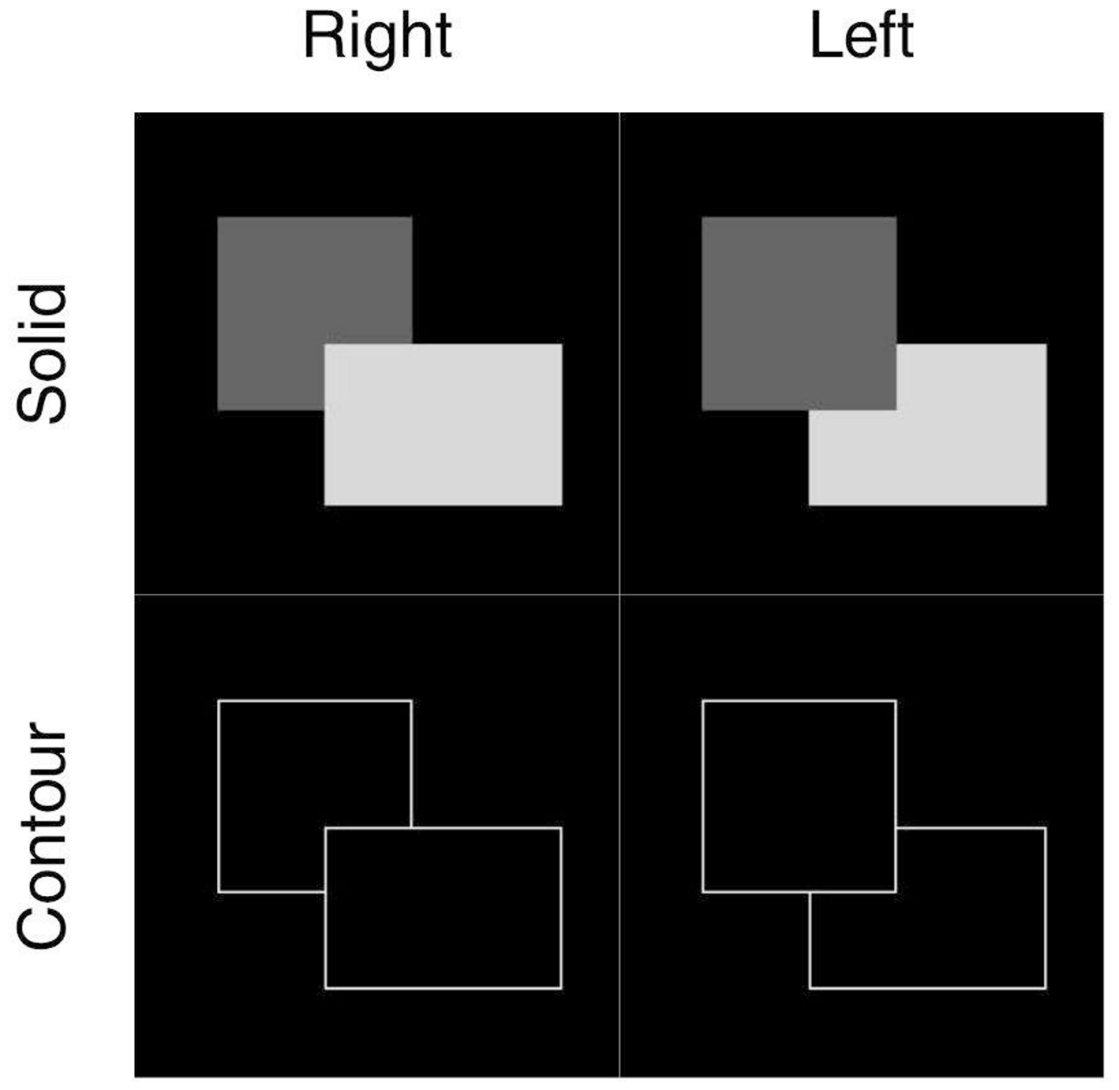
Examples of the two luminance-condition stimulus types. Rows represent stimulus types, with solid indicating filled in shapes of different luminance, and contour indicating outlines of occluding shapes. Columns provide examples of borders owned by right and left rectangles, respectively. The networks were trained to distinguish right vs. left BOS using these labels via supervised learning.

We rendered two luminance settings for the experiments as depicted in Figure 2. The luminance-invariant (contour) condition displayed rectangles as hollow outlines with their borders set to a high contrast value and thereby eliminated surface cues and forced reliance on border geometry for BOS. In luminance-variant (solid) condition, the two rectangles were filled at two grayscale levels such that:


$$l_{L},l_{R}\in \left[50,250\right],\left|l_{L}-l_{R}\right|\geq 50$$

Where $l_{L},l_{R}$ were the grayscale values for the rectangles. We kept an absolute difference to ensure a strong luminance contrast that would not trivially disappear at close luminance values.

Models were trained in a supervised fashion, with ground-truth classification labels assigned based on the occlusion relationship of the two rectangles for a specific stimulus. The rectangle in the foreground was labeled with ownership of the shared border and the background rectangle was considered occluded. Models had to learn to classify stimuli following this occlusion labeling.

#### 2.1.1 Fragmented stimuli

**Figure 3:**
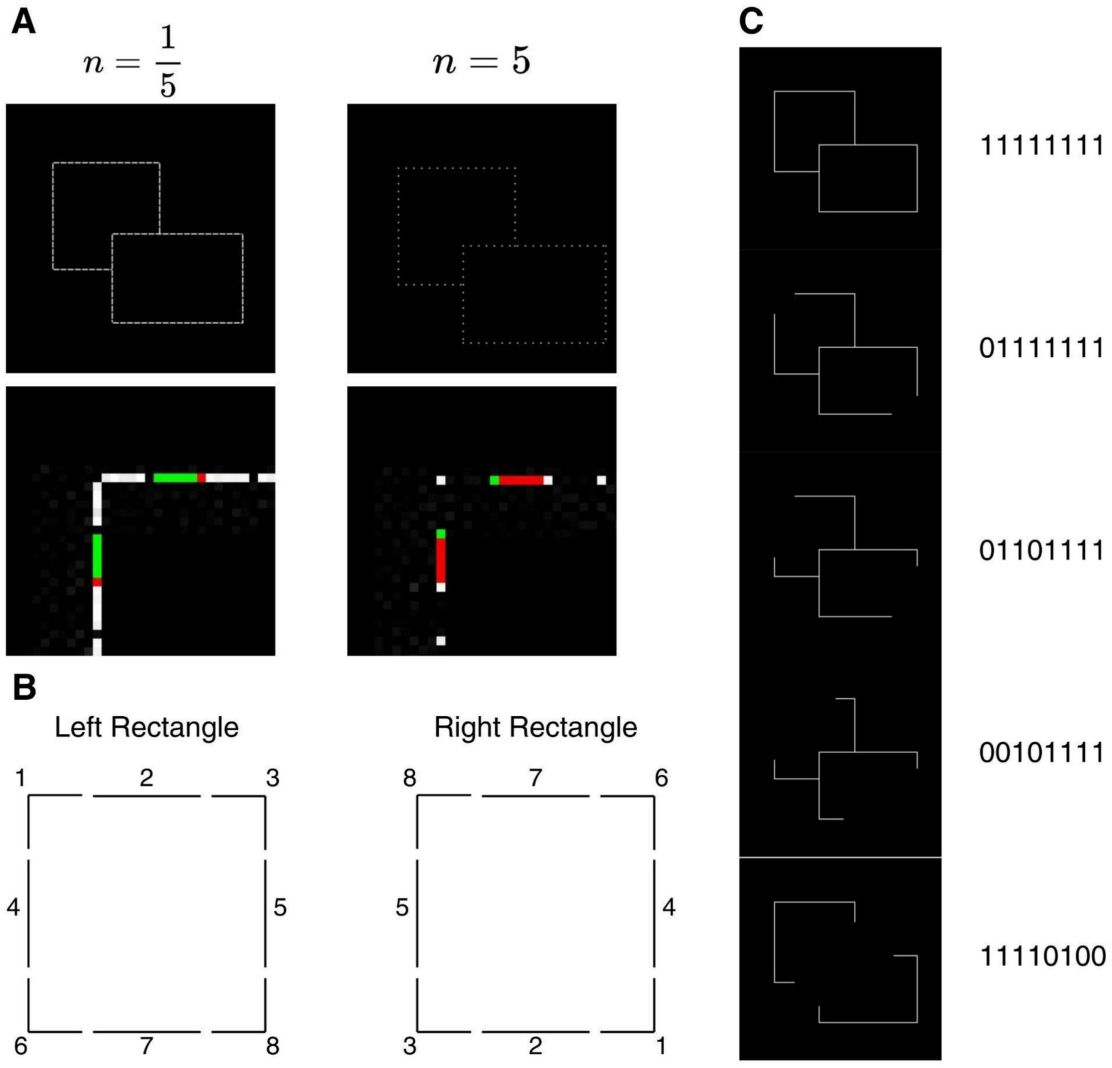
Fragmentation stimuli displays. **(A)** Example stimuli with two levels of gap. The parameter *n* represents a ratio between continuous contour and gap, forming dashes with spacing according to *n* value. For example, *n* = 5 means after every 5 pixels, one is visible and the rest are missing, whereas ⅕ means after each 5 sequential visible pixels there is one missing. The two examples are shown with *n* = ⅕ and 5, respectively. The upper row presents the full stimulus image, and the lower row shows a magnified view of the contour construction, where green pixels indicate the visible border segment and red pixels represent gap (missing) pixels. Variable gap levels were applied only to contour stimuli and not solid stimuli. **(B)** Positional fragment indexing for fragmented permutation of stimuli. Each number fragment corresponds to a different sector of the stimulus. **(C)** Example of fragmented stimuli represented by 8-bit binary codes, where each digit indicates the absence (0) or presence (1) of the corresponding fragment. The result of all 256 groups of stimuli are included in Supplementary Table 1.

We tested the robustness of the network by creating a series of testing datasets that were based on the contour stimulus (Figure 3). We tested network tolerance under fragmented border conditions, as objects in natural scenes are often displayed as discontinuous boundaries due to occlusion or fragmentation caused by shadow or texture. Nevertheless, BOS-selective neurons continue to respond when dealing with disrupted borders (Zhang and von der Heydt, 2010), which indicates the ability of biological vision to merge separate visual elements into single border representations. To probe whether CNNs exhibit the ability to preserve BOS assignment under fragmented conditions, we systematically designed two classes of fragmented figures to examine network performance.

For the first class of condition, we produced 10 groups of figures with discontinuous borders by dividing the contours of rectangles from the contour stimuli group into evenly spaced segments with increasing intervals (Figure 3A). We did not fragment the solid stimuli group since fragmenting filled shapes would introduce ambiguity about whether removed regions should be interpreted as holes or as changes in surface appearance rather than as missing contour information. The degree of interval was controlled by a border-to-gap ratio, ranging from 5 border pixels with 1 gap pixel ( $n=\frac{1}{5}$ ) to 1 border pixel with 5 gap pixels ( n=5 ). These figures were constructed using the same procedure as in the training set, but with their contours modified into dashed outlines.

For the second class, we used methods similar to Zhang and Von Der Heydt (2010), where the rectangle was divided into 8 fragments with each fragment either absent or present during testing. This factorial design created a total of 256 combinations. Fragments were paired diagonally between left and right rectangles since our stimuli generative model always placed the right rectangle at the bottom right quadrant relative to the left. Each fragment was subsequently assigned an index (Figure 3B). By doing this, we generated a unique 8-digit binary code for every combination. Specifically, the order of digits was represented by the numbering of fragments from Figure 3B with 1 being present and 0 being absent. For instance, 00101111 means that fragments 1, 2, and 4 were absent in the stimuli (Figure 3C).

### 2.2 Model

**Figure 4:**
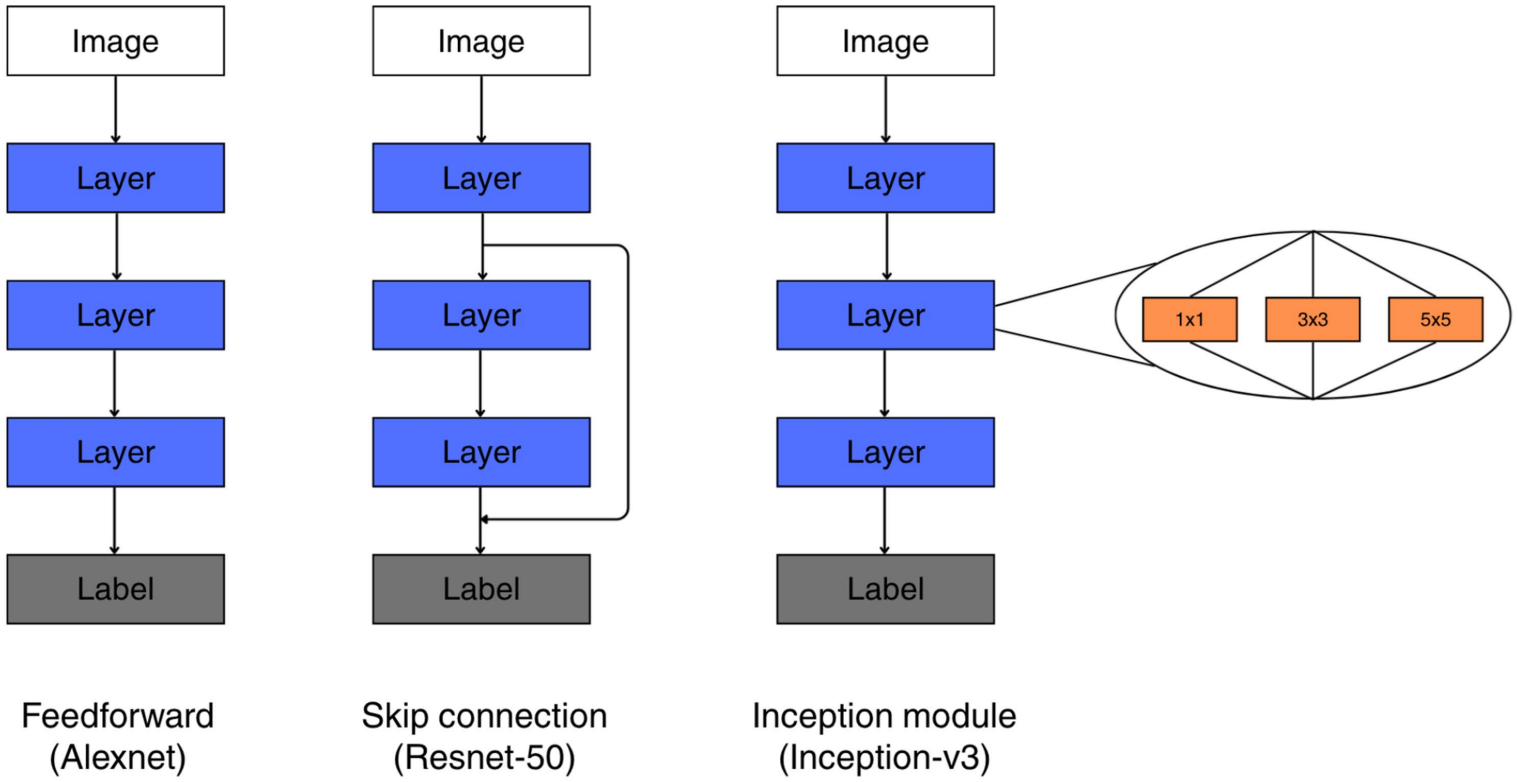
Illustration of the key architectural differences between the three network architectures evaluated. Layers refer to trainable convolutions. For inception, kernel sizes of parallel convolutions are denoted in orange. Labels refer to classification heads, consisting of trainable fully connected layers and sigmoid classification layers.

The CNNs are a class of deep learning models designed for image analysis. They process the images in order of layers by applying learned spatial filters to local regions of the input and nonlinear transformations. The local responses are cumulative as information moves through the network so that later layers can encode increasingly complex and spatially extended patterns. Compared to traditional computer vision systems, CNNs learn all filtering operations directly from data during training and optimize parameters through gradient-based optimization. In this work, three CNNs with different architectures were trained on the three versions of datasets (solid, contour, mix of solid/contour) with the objective to identify which shape owned the border between two overlapping ones. We used AlexNet to serve as our baseline model due to its relatively shallow structure and interpretable feature representation. To test whether adding residual learning or multiscale feature extraction perform better than purely feedforward, we chose Inception-v3 and ResNet-50, respectively, as alternate architectures (Figure 4).

For each stimulus group used in the cross-conditions (both the solid/contour and cross-shape), we randomly generated 20,000 images and partitioned them into training, validation, and testing subsets using a 7:1:2 ratio. The validation subset was used exclusively for hyperparameters tuning, while the testing subset was reserved for the final performance evaluation. Detailed training hyperparameters and loss functions for each model are provided in Supplementary Table 2. For the fragmentation conditions, which we used as a test set only for measuring robustness and not for network training, we randomly generated sets of 2,000 images per group (10 groups) for the first class of fragmentation (gap level) and 1,000 images per group (256 groups) for the second class of fragmentation (fragments).

#### 2.2.1 AlexNet

AlexNet is an early and relatively simple CNN structure that processes images through a sequence of convolutional and pooling layers arranged in a strictly feedforward manner. Information flows feedforwardly through the network, with each layer operating on the output of the previous one. Consequently, AlexNet is a comparatively shallow and structurally simple architecture by modern standards, which makes it a standard benchmark for examining how well purely one-to-one feedforward connections could account for BOS selectivity.

#### 2.2.2 Inception module

Compared to the traditional CNN structure that consists of combinations of convolution-nonlinearity-pooling, Inception networks extend the basic CNN framework by allowing multiple types of visual features to be extracted in parallel at each processing stage. It incorporates multiple parallel convolutional layers with different filter sizes and concatenates the resulting feature map at the end as a single joint representation (Szegedy et al., 2015). Therefore, instead of using a single filter size at each layer, Inception modules apply several convolutional filters of different spatial scales simultaneously and combine their outputs, enabling the network to capture both fine local details and broader spatial patterns within the same layer.

The entire module consists of four individual pathways, a pathway of single 1×1 convolution that performs channel recombination, two pathways of 1×1 convolution to reduce dimensions following by either a 3×3 or 5×5 convolution to integrate medium to large spatial pattern, and finally a 3×3 maxpooling to introduce translational invariance which is potentially significant as our stimuli have varying position in the image. With appropriate padding, the four pathways result in the same spatial dimension but different number of channels, making the final merge achievable.

#### 2.2.3 Residual connection

ResNet provides a solution to the degradation problem in deep networks by introducing residual (skip) connections that allow information to propagate from shallower layer to deeper layer without going through the intermediate layers. This design enables feature reuse and preserves low-level visual information, such as edges and corners, which are particularly critical for accurately processing our geometric stimuli and supporting reliable BOS decisions.

### 2.3 Analyzing performance

#### 2.3.1 Network accuracy

Considering the multiplicity of networks and training sets, we used the pairwise McNemar’s test (Spoerer et al., 2017) to compare classification accuracy between different networks trained on the same dataset. The non-parametric attribute makes it suitable for the evaluation of significant differences in their prediction outcomes when tested on the same datasets. To account for the multiple comparisons, *p*-values were adjusted using the Benjamini–Hochberg false discovery rate (FDR), with the FDR set to 0.05, to control the expected proportion of false positives.

For comparison across versions of an individual network, we implemented the two-proportion z-test to determine whether the performance achieved by a network trained on contour when tested on solid differs significantly from the performance achieved in the reverse direction. This thereby determined the generalization and robustness of the networks to other types of stimuli.

#### 2.3.2 Saliency mapping

In order to probe the internal decision-making process of the network and identify the features driving its predictions, we examined the saliency of the learned representations through Gradient-weighted Class Activation Mapping (Grad-CAM) that highlighted the most informative image regions for classification in a heatmap (Selvaraju et al., 2020). Through saliency mapping, we analyzed what image cues contribute to BOS representation and how important features evolved at different stack bundles of convolution-nonlinearity-pooling in the network hierarchy. Grad-CAM functions by calculating the target class score gradient with respect to the feature maps of the selected layer and averaging it to determine the importance of each channel. The weighted combination of feature maps is then rectified and upsampled to produce a class-discriminative heatmap that highlights the image regions most influential to the model’s prediction. This allowed visualization of how each network represents BOS-related cues and how these cues affect ownership decisions in different architectures and training scenarios.

## 3 Results

### 3.1 Quantitative analysis of fragmentation performance

#### 3.1.1 Generalization of network

**Figure 5:**
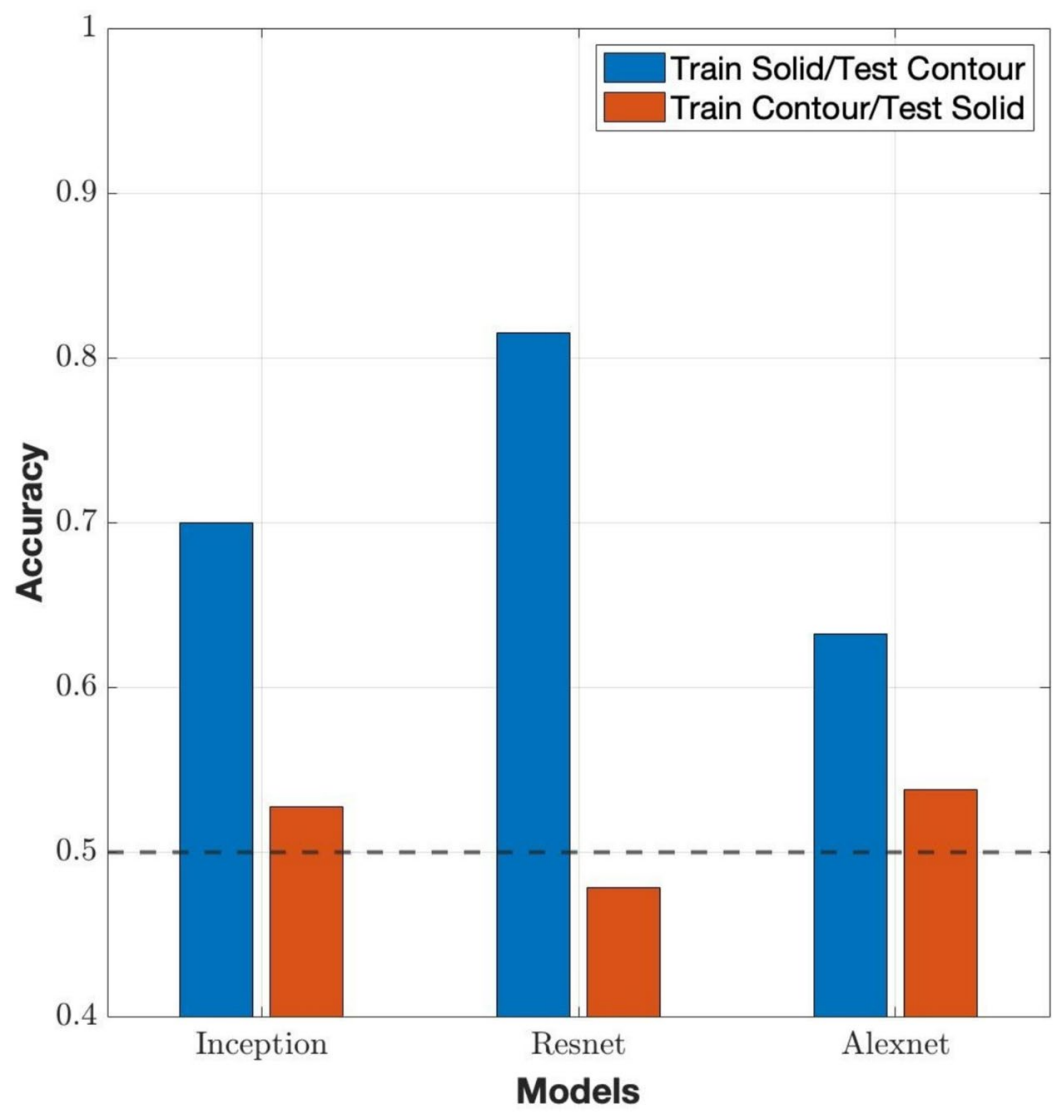
Classification accuracy of three different model architectures under solid/contour cross-condition evaluation. Permutations of models were trained on contour stimuli and tested on solid stimuli (train contour/test solid) and also trained on solid stimuli and tested on contour (train solid/test contour; see Figure 1 for examples of stimulus sets). Each model architecture performed around chance (50%, marked by the dashed line) when trained on contour stimuli and tested on solid stimuli, whereas accuracy was substantially higher when trained on solid stimuli and tested on contour.

The generalization was shown by networks trained under one luminance condition and tested under opposite conditions. All networks showed significant differences ( $p<0.001;z_{I}=6.03,z_{R}=22.26,z_{A}=11.23$ ) where networks trained on solid stimuli outperformed networks trained on contour stimuli (Figure 5). This implies that solid stimuli supplied more transferable information through combined contour and surface cues, whereas contour stimuli did not sufficiently prepare the network to interpret ownership when luminance-defined regions were introduced. Overall, the results demonstrated a strong directional dependence in generalization, with solid-trained networks transferring more effectively to contour conditions than contour-trained networks transfer to solid conditions.

**Figure 6:**
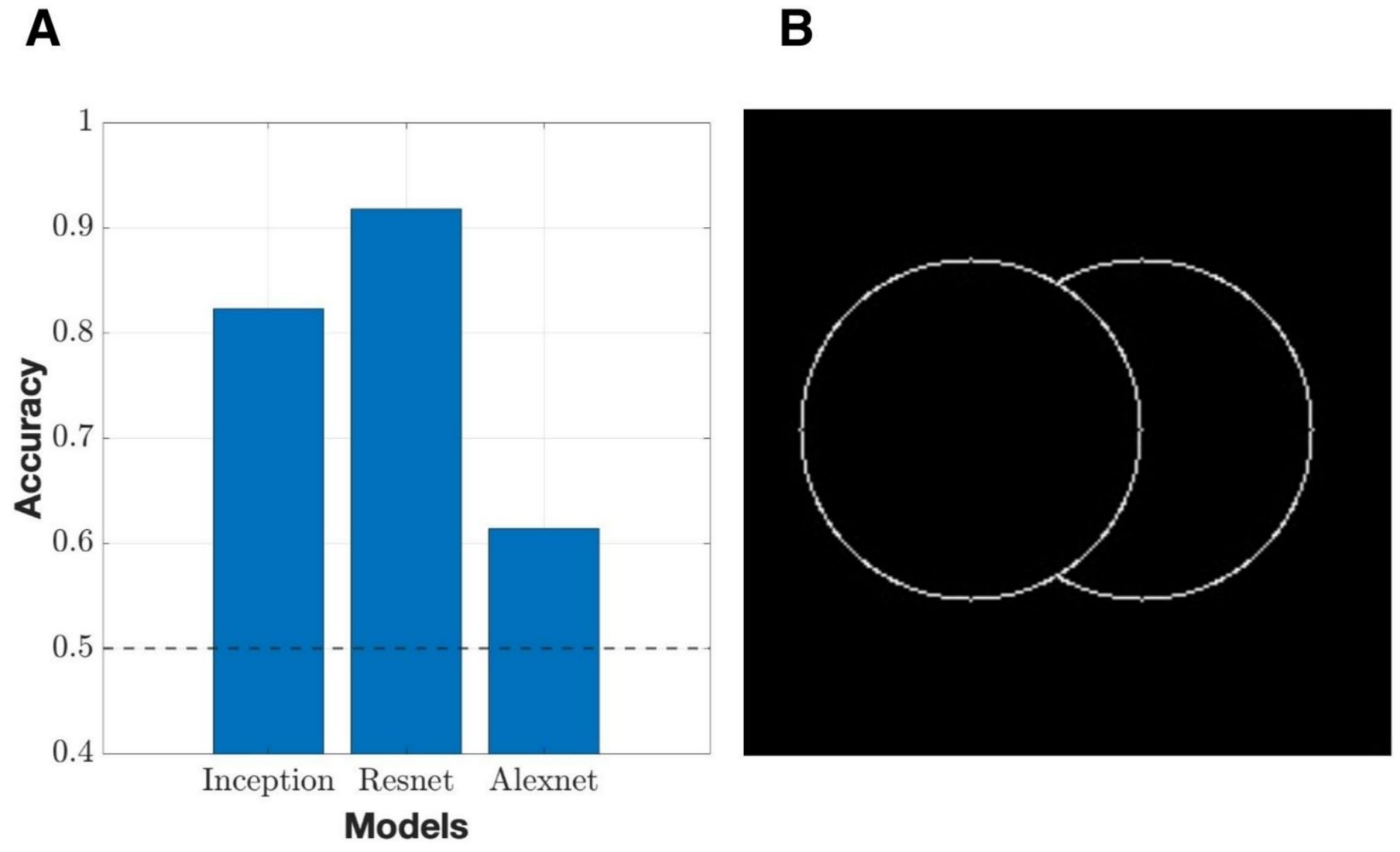
Cross-shape generalization from rectangles to circles. **(A)** Classification accuracy of three different network architectures trained on contour rectangle stimuli and tested on contour circle stimuli with analogous occlusion configurations. The dashed line indicates chance level (50%). All models achieve above-chance performance, with ResNet showing the strongest transfer and AlexNet the weakest. **(B)** Example of a left-owned test stimulus from the circular contour dataset, illustrating the geometric structure used in the cross-shape generalization experiment.

In addition to the luminance conditions, we also tested whether networks generalized beyond specific shapes by performing a cross-shape evaluation (Circle vs. Rectangle) in which all networks were trained on contour rectangles and tested on contour circles with similar occlusion relationships. Despite the networks never having been exposed to other geometric stimuli, all networks were able to retain above-chance (50%) accuracies (Figure 6). This in part indicates that the learned representations did not depend on the general geometry of the object, but rather on more shape-invariant cues that signal BOS, such as junction configuration and relative contour arrangement. The accuracy of the three networks also followed a trend in line with their accuracies observed in the solid/contour cross-condition.

#### 3.1.2 Performance across networks

**Figure 7:**
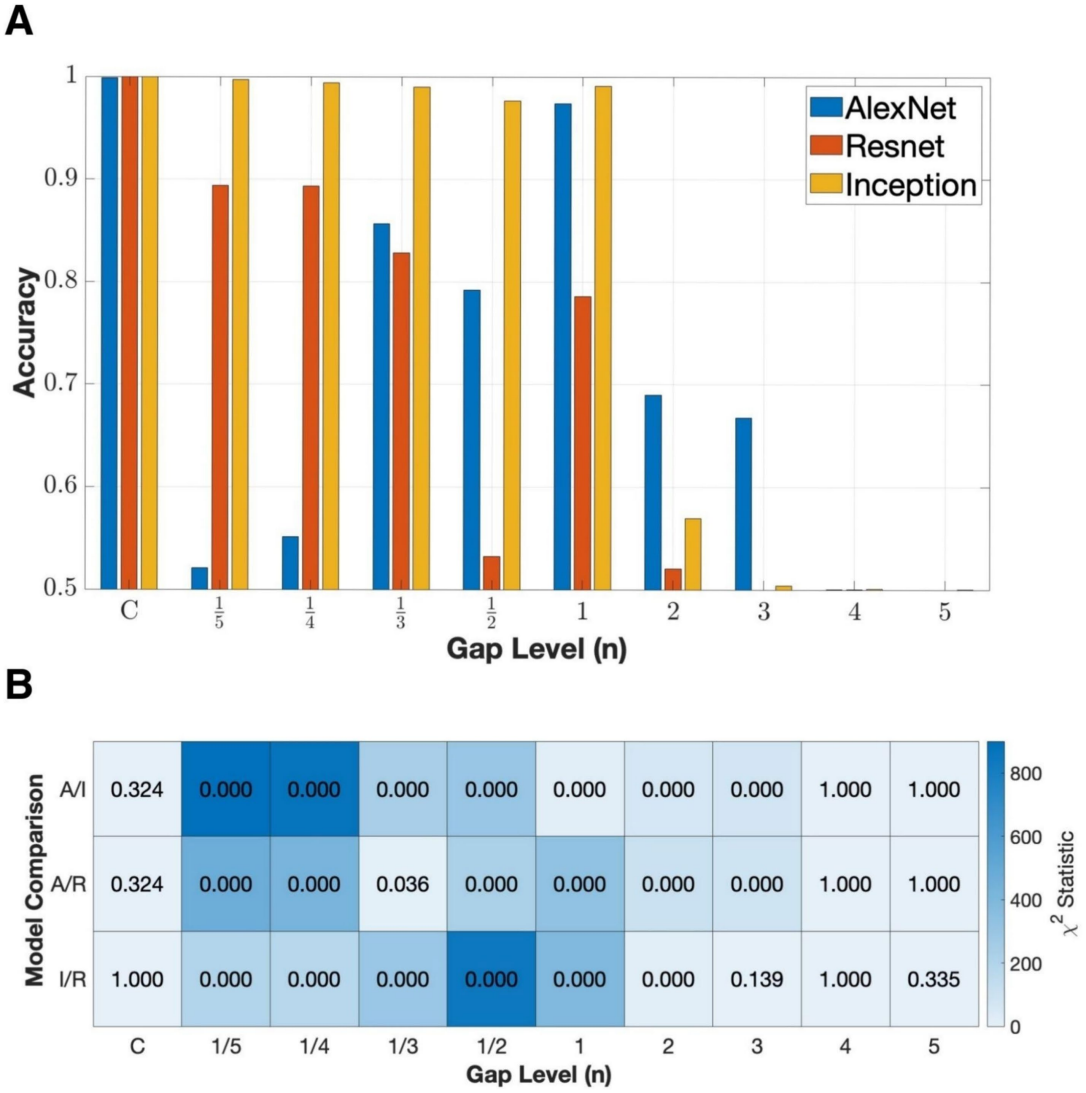
Network performance under incremental gap levels. C represents the contour stimuli with no gap, with the gap parameter progressively indicating exaggerated dashing of the contours of the stimuli (see Figure 3A for example). **(A)** Classification accuracy across increasing gap levels. **(B)** Pairwise McNemar’s test result comparing model accuracy at each gap level (A/I: AlexNet vs. Inception, A/R: AlexNet vs. ResNet, I/R: Inception vs. ResNet). Color intensity indicates the $\chi^{2}$ statistics, and each cell displays the FDR-adjusted *p*-value for the corresponding pairwise comparison.

The networks were trained using contour rectangles with continuous borders to recognize discontinuous borders with increment gaps to test the robustness and generalization of different feedforward structures (Figure 3A). Within the small gap ( n≤1 ) conditions, Inception performed the best and exhibited strong robustness with consistent high accuracies above 95% and significant differences with both ResNet and AlexNet (Figure 7). ResNet performed moderately well in this regime, with accuracies near 90%, but still significantly below Inception for nearly every gap size. AlexNet showed an irregular fluctuation where the steepest degradation happened as even modest discontinuities were introduced but rose again at larger gaps, though recovery was unstable. As gap levels increased ( n≥1 ), all networks showed progressive decline in performance. The lack of significance at extreme gap level ( n≥4 ) indicates that all networks failed under highly discontinuous contours, consistent with the absence of sufficient edge information for reliable BOS assignment.

The contrast between the performance of Inception under fragmentation (Figure 7) and in the solid/contour cross-condition (Figure 5) demonstrates that the two generalizations, i.e., across fragmentation and across solid/contour, were different. Fragmentation maintains contour-based representation and simply removes portions of the stimulus, whereas solid/contour cross-condition generalization requires the network to bridge between qualitatively different cue domains. Inception’s multiscale structure appeared to be well suited for integrating incomplete contour information, but less effective at generalizing across contour vs. solid-based representations.

#### 3.1.3 Fragment contributions and interaction effects

To evaluate the significance of individual fragments and their interaction, we used a second-order regression model similar to Zhang and Von Der Heydt (2010) but without the contrast polarity term as follows:


$$R=\beta_{0}+\sum_{i}d_{i}F_{i}+\sum_{i<j}f_{ij}F_{i}F_{j}+g\left(N\right)$$

Where R is the accuracy of the network, $\beta_{0}$ is the baseline accuracy of the network and a fitted intercept of all conditions, $F_{i}$ and $F_{j}$ are fragment variables (0 when absent; 1 when present). $d_{i}$ and $f_{ij}$ are regression coefficients that estimate the significance of individual fragment i and the interaction between two fragments in the BOS decision. g(N) is a normalization function that eliminates the global effect of fragment availability N in a given stimulus.

**Figure 8:**
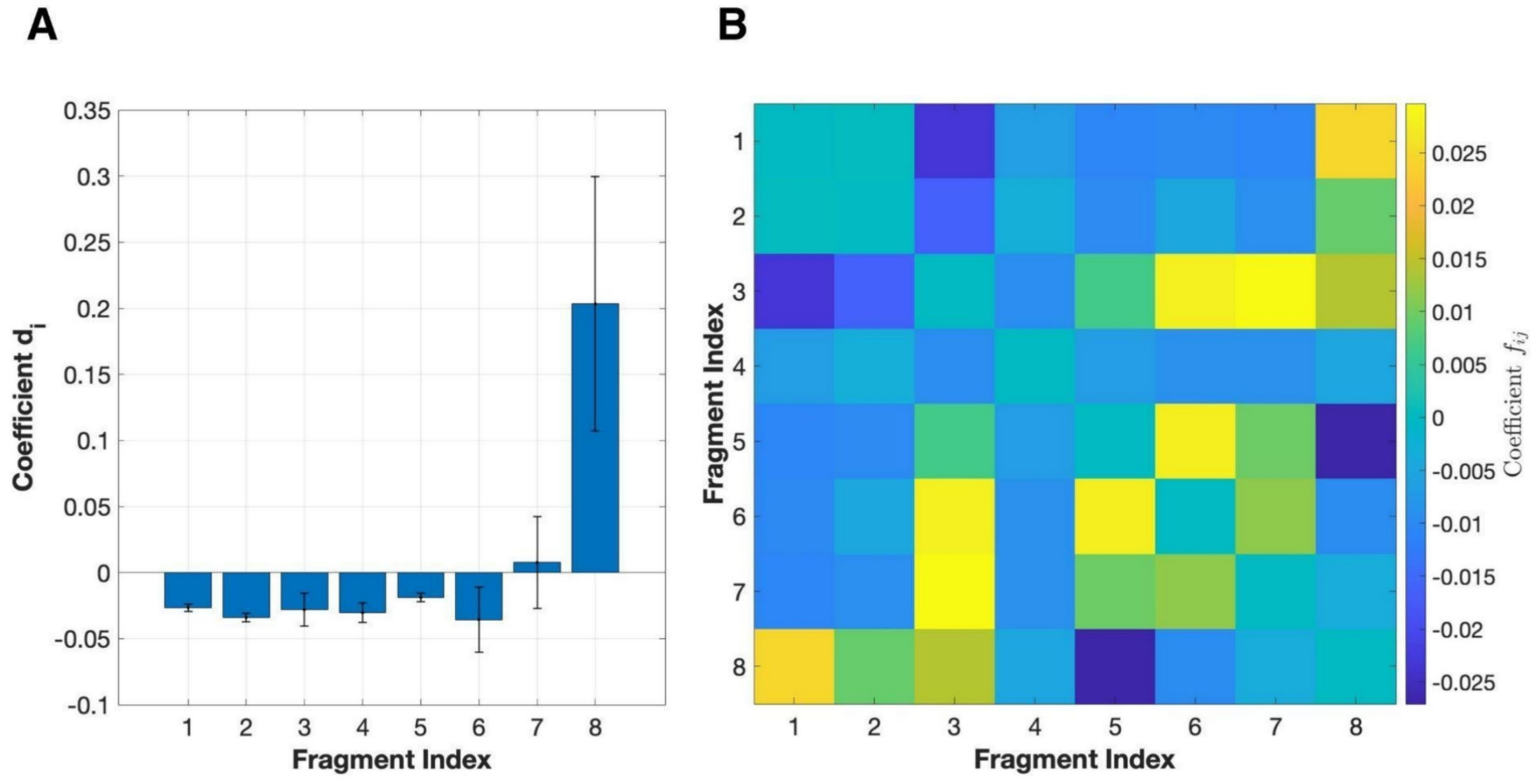
Fragment contributions and interaction effects estimated by the regression model. **(A)** Illustration of individual fragments (Figure 3B) averaging across networks. **(B)** Matrix of pairwise interaction coefficient $f_{ij}$ . A high $f_{ij}$ implies a synergistic effect in BOS decision performance for that pair of fragments.

The model revealed a highly structural pattern in terms of how networks perform BOS where the individual fragment coefficient displayed a sharply unequal distribution of significance. Among eight of the fragments (Figure 8A), only fragment 8 exhibits a strong positive effect on BOS decision. In contrast, the remaining seven fragments showed only small negative or near-zero effects, suggesting that they contribute little individually and even present local ambiguity when present alone. This asymmetric pattern implies that the networks rely selectively on specific, spatially critical fragments rather than treating all boundary segments equivalently. The fragment interaction coefficient further clarified this pattern (Figure 8B). Several fragment pairs exhibited strong positive interaction (1–8, 3–7, 3–6, 5–6), demonstrating a synergy effect in which BOS information became more complete and obvious to the network when these fragments existed together than independently. This interaction likely reflects the grouping mechanism in biological vision that relies on a certain combination of elements for recognition. Conversely, there were some fragment pairs that interfered with the network’s decision (1–3, 5–8) by introducing confounding or opposite structural cues about BOS.

To account for the influence of global fragment counts, we introduced a normalization function that adjusted for the general improvement in the model’s accuracy as more fragment information was presented in the stimulus.


$$g\left(N\right)=\gamma_{1}N+\gamma_{2}N^{2}+\beta_{0}$$

**Figure 9:**
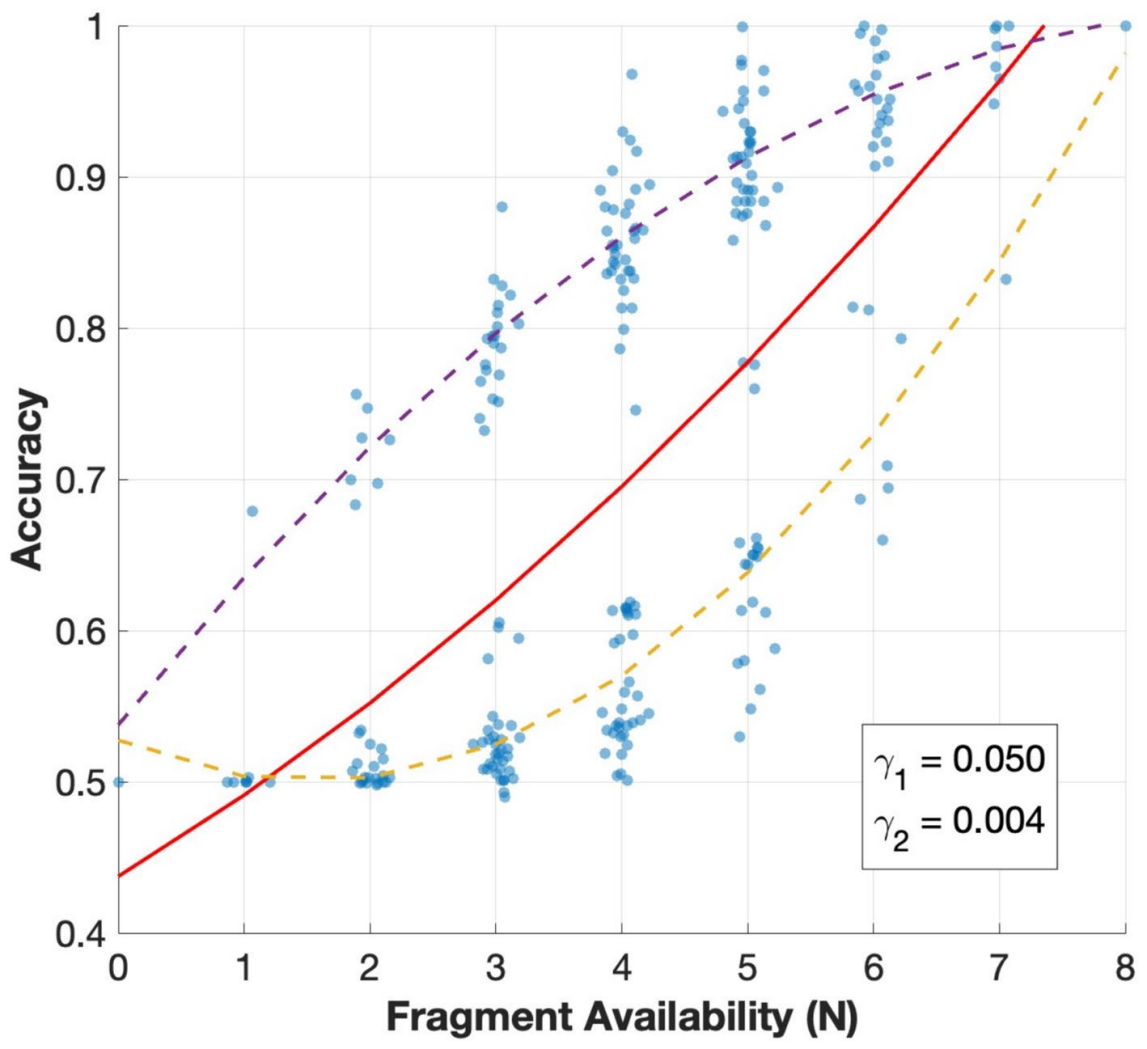
Effect of fragment availability (*N*) on classification accuracy. Fragment availability refers to the number of fragments present in the stimulus, irrespective of their position. For each number of fragments available, all permutations of fragment positions were tested, with each point corresponding to the accuracy for a single stimulus condition with a specific fragment configuration (Figure 3C). Points are grouped by the fragment availability with a small horizontal jitter applied for visualization, and the red curve shows the fitted normalization function with linear coefficient $\gamma_{1}=0.050$ and quadratic coefficient $\gamma_{2}=0.004$ . The dashed purple and yellow curves represent separate regression analyses for stimuli groups in which fragment 8 is present versus absent, respectively.

Where the linear coefficient $\gamma_{1}$ represents the improvement from adding fragments regardless of their specific location. The nonlinear coefficient $\gamma_{2}$ captures the curvature of this relationship, expressing the degree of saturation or acceleration as more fragments are added. From the simulation, the two coefficients indicated a consistently positive effect of fragment availability (Figure 9). The large linear term indicated that each additional fragment increased accuracy on average, and the small quadratic term suggested that this improvement did not saturate within the tested range. Instead, the network continued to benefit from incremental fragment cues even at high fragment availability. The observed bimodal distribution arose primarily from the strong contribution of fragment 8, whose presence or absence largely determined whether sufficient occlusion-specific structure was available to support a reliable BOS decision.

### 3.2 Saliency-based analysis

#### 3.2.1 Saliency across network hierarchy

**Figure 10:**
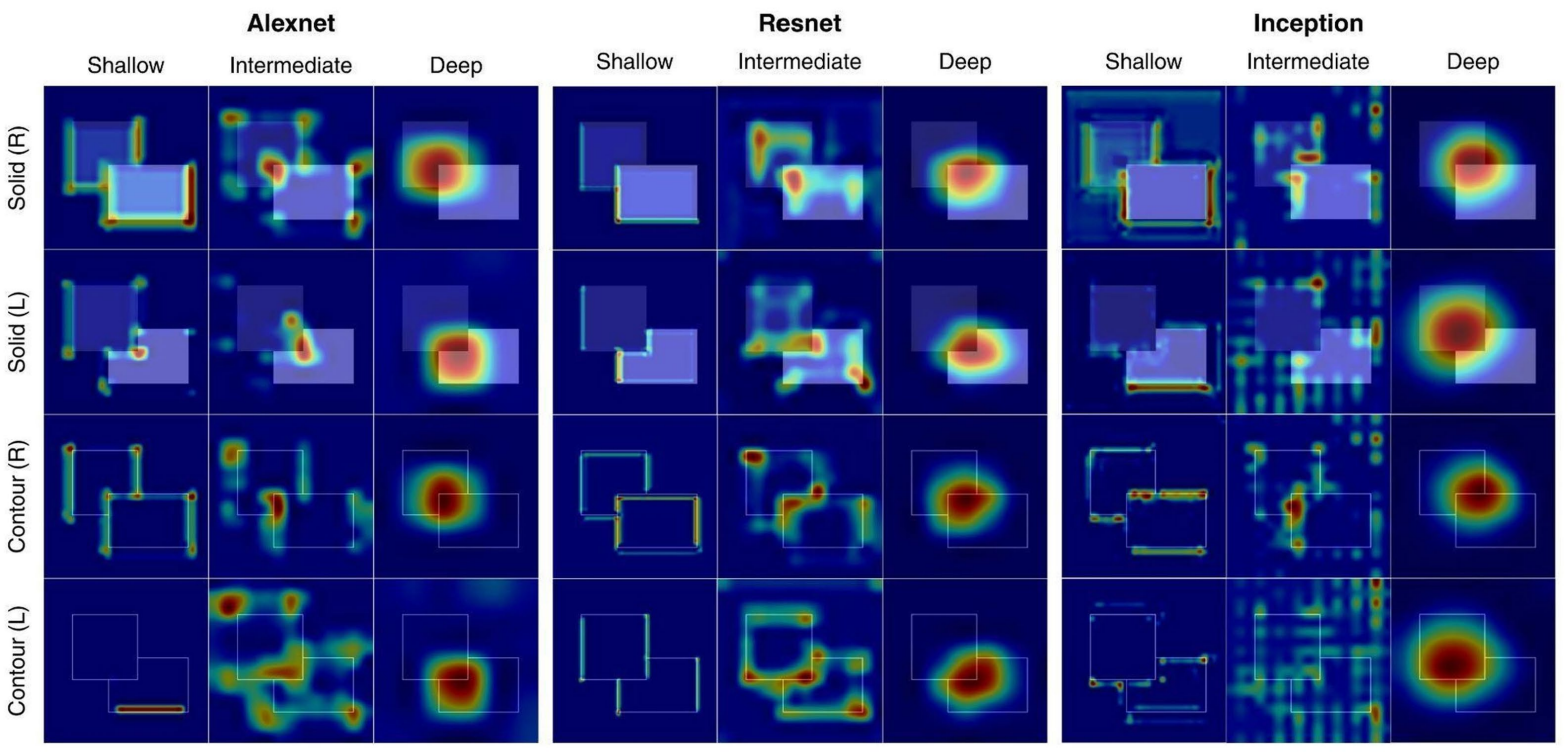
Visualization of Grad-CAM across network architectures and layer depth (AlexNet: *pool1/pool2/pool5*; ResNet: *add1/add8/add16*; Inception: *mixed1/mixed5/mixed10*). Heatmaps reflect the most salient regions of stimuli to the network’s BOS classification. In shallow layers, networks appeared to focus on low level features such as edges, but in deeper layers, saliency was refined to T-junctions and corners (L-junctions). All networks evaluated were trained on mixed datasets consisting of both contour and solid stimulus patterns. *PoolK, addK, mixedK* are standing for networks’ layers – higher *K* means deeper layer.

Across all three networks that trained on a mixed dataset, Grad-CAM visualizations showed a clear hierarchical progression in how BOS information was represented and evolved (Figure 10). Shallow layers were more responsive to low-level visual elements such as edges, corners (L-junctions), and isolated contour fragments, generating saliency patterns that were spatially scattered and not yet aligned with the ownership-defining border. Intermediate layers began to integrate these local features into larger spatial structures, showing more continuous activation along contour segments and increased sensitivity to the relative arrangement of the two rectangles. By the deep layers, the saliency converged into a compact, highly localized region centered on the occluding area, indicating that ownership decisions were made at later stages of processing, and the activation patterns consistently corresponded to the correct ownership boundary regarding the stimulus type. Overall, these results demonstrate that BOS selectivity is a hierarchical computation that gradually evolves from distributed local feature detection to a coherent, border-specific representation in deeper stages of the network.

#### 3.2.2 Saliency across fragmentation

**Figure 11:**
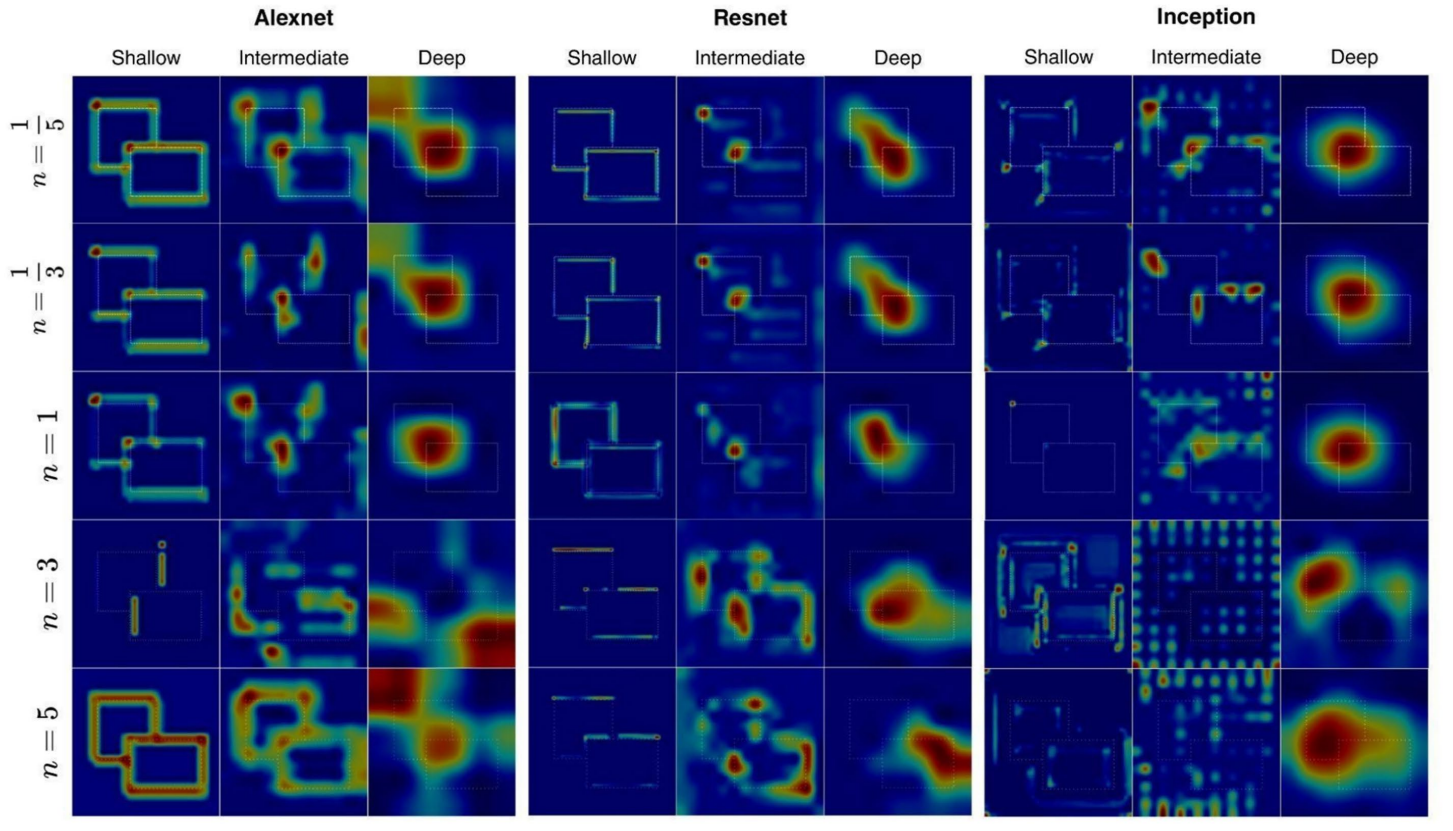
Visualization of Grad-CAM across increment gap levels (*n* ∈ {⅕, ⅓, 1, 3, 5}, see Figure 2). Heatmaps reflect the most salient regions of stimuli to the network’s BOS classification, essentially highlighting the regions of stimuli most indicative of the BOS relationship with respect to different depths of the network. Saliency tended to become less specific as gap increment (*n*) increased. All networks evaluated were trained on contour datasets with no gap.

For fragmentation, Grad-CAM visualizations showed a gradual loss of and reorganization of BOS representations with increasing gap level (Figure 11). For small gaps, saliency strongly aligned with the fragmented contour segments that together defined the owned border, suggesting that the networks captured discontinuous local evidence and assembled this into a coherent ownership signal. As gap size grew, saliency became less continuous and distributed more unevenly among the remaining contour fragments. At very large gap levels, however, saliency became spatially non-specific, especially at deep layers where saliency was more spread out and shifted toward regions other than those occluded. Under these conditions, the deep layer activation patterns ceased to be coherently associated with the true ownership boundary, and correspondingly the classification accuracy dropped sharply even when early layer saliency remained consistent with that observed for intact contours. In general, the representation of BOS in CNNs degrades with increasing fragmentation, as we see a transition from continuous contour-based representations to the sparse and unstable cues lacking in power to assign ownership reliably.

## 4 Discussion

We trained various hierarchical architectures of CNNs to perform basic BOS and used explainability methods to interrogate the stimulus features underpinning their ownership classification. This study shows how BOS is developed by generalization *in silico* hierarchical feedforward visual networks, granting insight into feedforward edge/junction detection mechanisms critical to BOS which could be analogous to biological systems. By examining where the performance of feedforward architectures degraded, we can gain insight into the contribution of feedback connections in biological systems.

### 4.1 Hierarchical BOS representation

The CNN architectures tested showed a hierarchical pattern of BOS-related information representation according to saliency analysis. Shallow layers mainly responded to edges, with intermediate layers focused on extended contour patches, corners, and junction structure, while deep layers generated compact and spatially coherent patterns of activation that mapped closely to the occlusion region rather than to other individual features. This progression was consistent among stimulus types and architectures, suggesting that it embodies a general attribute of hierarchical CNN architecture processing.

This pattern mirrors the organization of the primate visual system, where receptive fields expand in size and contextual modulation increases along the ventral stream (Felleman and Van Essen, 1991; Angelucci and Bressloff, 2006). Specifically, occlusion-centered saliency emerged in deep layers, implying that instead of just the explicit border encoding, CNNs create ownership representations that signify a more global interpretation of figure–ground structure. These representations are analogous to those incrementally higher visual areas, where BOS signals are able to accommodate local variations in edge locations, while sensitivity to overall object configuration is still present (Hesse and Tsao, 2016).

However, this hierarchical emergence occurred in all network structures even without explicit recurrent or feedback connections, which are critical for processing biological BOS (Layton et al., 2012, 2014). This indicates that feedforward processes are adequate to generate BOS-like representations robust to different stimuli fragmentation and gap conditions, but they do not imply mechanistic equivalence with the cortical circuits. This does not reject the notion that feedback can also contribute to further consolidation of BOS representation. Rather, our results clarify which aspects of BOS can be represented by feedforward processes and which likely require additional circuitry.

### 4.2 Role of T-junction cues

The regression analysis (Figure 8A) revealed that BOS decisions made by the networks are highly impacted by fragment 8. This fragment was spatially located at the corner of the overlapping region between the two rectangles and, together with its paired counterpart, formed a T-junction configuration characteristic of occlusion geometry. The disproportionately larger and significant individual fragment coefficient associated with it suggests that networks assign substantially greater importance to this occluding central feature than others when inferring and representing ownership. This is supported by the saliency map, as the deep layer activations are consistently concentrated on this fragment across stimuli and networks.

These results are similar to those reported by Zhou et al. (2000), which demonstrated that BOS-selective neurons respond most robustly at regions that participate in the T-junction. Unlike individual edges, T-junctions determine BOS by indicating the geometry of the occlusion, rather than relying solely on orientation, which makes it one of the most reliable monocular cues for depth ordering and figure–ground segregation in biological vision. The configuration of T-junctions also provides a basis for ownership inference across changes in object size because their geometric patterns remain unchanged during scaling operations. Occlusion cues based on junction geometry have been shown to support figure–ground segregation across variations in projected retinal size when relative contour support and alignment are maintained (Lesher, 1995).

As such, the dominance effect of occluding fragments implies that the network has developed a preference for junction-like and overlapping configurations and incorporates a biological-proven BOS cue that enables size-invariance of representing occlusion during supervised learning.

### 4.3 Fragment integration and gestalt principle in BOS

Aside from individual fragment effects, the performance of BOS was also importantly influenced by the manner in which contours were arranged continuously. Our findings showed that BOS accuracy was stable up to gap level *n* = 1, i.e., every other pixel visibility (Figure 7). Under these circumstances, networks can integrate edge information across small gaps to have a coherent representation of the ownership border. The networks could hold on to significant BOS representation up to gap level *n* = 1, and partially up to *n* = 2,3, where borders in the stimulus are fragmented into dot-like segments. This transition indicated a failure of networks to maintain perceptual continuation. Conventional Gestalt concepts suggest that collinear or smoothly oriented elements are more likely to be clustered into a single contour, whereas spatially separated points lack sufficient structure to support grouping (Field et al., 1993). In other words, the performance in contour integration will degrade dramatically when elements lose adequate continuation cues even while the local orientation information is preserved.

With respect to this view, the early layer saliency map in our network continues to pick up edges and corners even when contour gap is greater, but the local signal does not carry through to stable BOS representations at deeper stages, meaning the performance reduction is not caused by a weakened edge detection, but by network’s inability to perform global integration. In biological vision, it is believed that long-range horizontal and feedback connections influence the completion of contours and perceptual clustering when resources are constrained and make it possible for figure–ground signals to persist despite fragmented input (Angelucci and Bressloff, 2006; Gilbert and Li, 2013). Lack of such mechanisms in purely feedforward networks like those tested most likely makes them vulnerable when contour continuity no longer supports Gestalt grouping.

With regard to the effect of fragment availability, the networks exhibit a strong linear and weak quadratic relationship with the number of presence fragments (Figure 9). This trend is in accordance with the data from Zhang and von der Heydt (2010), which reported a positive correlation between contextual fragment availability and response magnitude among BOS-selective neurons, suggesting that more contextual data reinforces the signaling of BOS. However, there is a significant difference in the response profile: whereas the biological data shows a rapid increase followed by clear saturation as fragment availability increases, the networks of our study show more linear dependence and minimal signs of saturation.

## 5 Limitations and future directions

The CNN architectures tested clearly showed hierarchical patterns of BOS representation mirroring that of biological visual systems. It is important to note the simplicity of the stimuli used to train the networks to distinguish BOS, it is possible that the lack of feedback and horizontal processing would create more mechanistic divergence between CNNs and biological systems. We found that, whereas the biological data shows clear saturation as contextual information increases in stimuli, the networks of our study show more linear dependence and minimal signs of saturation.

Training networks on more complex BOS stimuli with more varied objects and junction geometries, or even natural images and stimuli, could reveal how more realistic BOS tasks benefit more from the additional connectivity in biological systems through more difficult to interpret contextual cues. Models may have accomplished the BOS task through the representation of depth, occlusion, or BOS between shapes in the stimulus, or via other means that are correlated to BOS. While this ambiguity is not unique to CNNs, as electrophysiological studies have likewise relied on simplified geometric stimuli that may also conflate BOS with closely related visual cues (Zhou et al., 2000), training with more complex stimuli could strengthen the interpretation of Grad-CAM saliency maps as visualizations of stimuli regions strictly related to BOS. To enrich our understanding of differing contextual requirements between CNNs and biological systems, models could be tested with other mechanisms of fragment removal or change (such as fragment rotation) to examine precise impacts of availability of visual context with regards to continuity.

Our proposed networks have merely feedforward processing, while biology is equipped with feedback among other mechanisms. These additional processes could aid in the corroboration of contextual information, facilitating increased certainty of BOS representation with less information. Hybrid CNN-RNN (recurrent neural network) models exist with recurrent connections approximating biological feedback but have not been extensively evaluated beyond their performance in basic image recognition benchmarks (Liang and Hu, 2015; Spoerer et al., 2017). Interrogating these architectures would allow for direct comparison with purely feedforward systems, possibly providing direct insight into the differences in BOS processes arising from these fundamental differences in connectivity.

In addition, disparities in performance and representational robustness across networks could be due to the variations in the number of layers, total number of learnable parameters, and architectural motifs. For instance, the superior accuracy and more stable saliency pattern of Inception (50 layers) during increment gap levels could arise from simply having more layers than AlexNet (8 layers). A more reliable approach would be to constrain the confounding characteristics and parameters while keeping training data constant. Exploring this issue could clarify whether the advantages observed are due mainly to the application of architectural principles (e.g., multiscale integration) or just increased network depth.

## Data Availability

The datasets presented in this study can be found in online repositories. The names of the repository/repositories and accession number(s) can be found in the article/Supplementary material.
