# Supplementary material for: Approaching human visual perception through AI-based representation of figure-ground segregation
Source: Front Psychol. 2026 Feb 27;17:1768533. doi: 10.3389/fpsyg.2026.1768533 (PMC12982451; doi:10.3389/fpsyg.2026.1768533)
Supplement: Supplementary file 1 [file Table_1.docx]

***Supplementary Material***

Generation code and datasets used to train the models are available in the linked

GitHub repository: <https://github.com/adenyip/bos-cnn-figure-ground>

**1. Supplementary Data**

| **Group ID** | **Binary Code** | **Fragment Availability (N)** | **Alexnet** | **Inception** | **Resnet** |
| --- | --- | --- | --- | --- | --- |
| 1 | 00000000 | 0 | 0.498 | 0.5 | 0.5 |
| 2 | 00000001 | 1 | 0.591 | 0.679 | 0.69 |
| 3 | 00000010 | 1 | 0.579 | 0.5 | 0.499 |
| 4 | 00000011 | 2 | 0.724 | 0.756 | 0.814 |
| 5 | 00000100 | 1 | 0.511 | 0.5 | 0.501 |
| 6 | 00000101 | 2 | 0.65 | 0.683 | 0.724 |
| 7 | 00000110 | 2 | 0.601 | 0.5 | 0.501 |
| 8 | 00000111 | 3 | 0.761 | 0.822 | 0.799 |
| 9 | 00001000 | 1 | 0.552 | 0.5 | 0.5 |
| 10 | 00001001 | 2 | 0.661 | 0.726 | 0.791 |
| 11 | 00001010 | 2 | 0.615 | 0.532 | 0.535 |
| 12 | 00001011 | 3 | 0.846 | 0.815 | 0.872 |
| 13 | 00001100 | 2 | 0.562 | 0.522 | 0.527 |
| 14 | 00001101 | 3 | 0.775 | 0.801 | 0.849 |
| 15 | 00001110 | 3 | 0.685 | 0.595 | 0.573 |
| 16 | 00001111 | 4 | 0.908 | 0.895 | 0.911 |
| 17 | 00010000 | 1 | 0.5 | 0.501 | 0.5 |
| 18 | 00010001 | 2 | 0.603 | 0.7 | 0.792 |
| 19 | 00010010 | 2 | 0.586 | 0.499 | 0.503 |
| 20 | 00010011 | 3 | 0.7 | 0.793 | 0.873 |
| 21 | 00010100 | 2 | 0.507 | 0.5 | 0.499 |
| 22 | 00010101 | 3 | 0.666 | 0.732 | 0.793 |
| 23 | 00010110 | 3 | 0.636 | 0.505 | 0.507 |
| 24 | 00010111 | 4 | 0.753 | 0.865 | 0.865 |
| 25 | 00011000 | 2 | 0.514 | 0.501 | 0.514 |
| 26 | 00011001 | 3 | 0.67 | 0.753 | 0.862 |
| 27 | 00011010 | 3 | 0.633 | 0.517 | 0.575 |
| 28 | 00011011 | 4 | 0.832 | 0.833 | 0.894 |
| 29 | 00011100 | 3 | 0.56 | 0.528 | 0.546 |
| 30 | 00011101 | 4 | 0.798 | 0.813 | 0.876 |
| 31 | 00011110 | 4 | 0.69 | 0.619 | 0.634 |
| 32 | 00011111 | 5 | 0.924 | 0.892 | 0.926 |
| 33 | 00100000 | 1 | 0.515 | 0.503 | 0.5 |
| 34 | 00100001 | 2 | 0.667 | 0.747 | 0.772 |
| 35 | 00100010 | 2 | 0.596 | 0.534 | 0.548 |
| 36 | 00100011 | 3 | 0.799 | 0.88 | 0.872 |
| 37 | 00100100 | 2 | 0.553 | 0.525 | 0.511 |
| 38 | 00100101 | 3 | 0.722 | 0.832 | 0.805 |
| 39 | 00100110 | 3 | 0.687 | 0.605 | 0.554 |
| 40 | 00100111 | 4 | 0.883 | 0.968 | 0.902 |
| 41 | 00101000 | 2 | 0.569 | 0.51 | 0.501 |
| 42 | 00101001 | 3 | 0.722 | 0.81 | 0.849 |
| 43 | 00101010 | 3 | 0.672 | 0.602 | 0.582 |
| 44 | 00101011 | 4 | 0.903 | 0.93 | 0.928 |
| 45 | 00101100 | 3 | 0.632 | 0.581 | 0.532 |
| 46 | 00101101 | 4 | 0.847 | 0.924 | 0.909 |
| 47 | 00101110 | 4 | 0.78 | 0.746 | 0.642 |
| 48 | 00101111 | 5 | 0.981 | 0.999 | 0.989 |
| 49 | 00110000 | 2 | 0.519 | 0.503 | 0.496 |
| 50 | 00110001 | 3 | 0.631 | 0.79 | 0.847 |
| 51 | 00110010 | 3 | 0.605 | 0.53 | 0.545 |
| 52 | 00110011 | 4 | 0.749 | 0.904 | 0.912 |
| 53 | 00110100 | 3 | 0.549 | 0.534 | 0.509 |
| 54 | 00110101 | 4 | 0.681 | 0.855 | 0.855 |
| 55 | 00110110 | 4 | 0.678 | 0.615 | 0.565 |
| 56 | 00110111 | 5 | 0.841 | 0.97 | 0.947 |
| 57 | 00111000 | 3 | 0.551 | 0.521 | 0.511 |
| 58 | 00111001 | 4 | 0.695 | 0.838 | 0.899 |
| 59 | 00111010 | 4 | 0.682 | 0.611 | 0.622 |
| 60 | 00111011 | 5 | 0.881 | 0.943 | 0.956 |
| 61 | 00111100 | 4 | 0.658 | 0.594 | 0.561 |
| 62 | 00111101 | 5 | 0.839 | 0.923 | 0.933 |
| 63 | 00111110 | 5 | 0.785 | 0.776 | 0.715 |
| 64 | 00111111 | 6 | 0.99 | 0.995 | 0.993 |
| 65 | 01000000 | 1 | 0.526 | 0.5 | 0.501 |
| 66 | 01000001 | 2 | 0.594 | 0.697 | 0.807 |
| 67 | 01000010 | 2 | 0.581 | 0.503 | 0.528 |
| 68 | 01000011 | 3 | 0.696 | 0.787 | 0.868 |
| 69 | 01000100 | 2 | 0.519 | 0.499 | 0.504 |
| 70 | 01000101 | 3 | 0.602 | 0.74 | 0.827 |
| 71 | 01000110 | 3 | 0.592 | 0.501 | 0.522 |
| 72 | 01000111 | 4 | 0.702 | 0.864 | 0.871 |
| 73 | 01001000 | 2 | 0.502 | 0.5 | 0.505 |
| 74 | 01001001 | 3 | 0.608 | 0.765 | 0.881 |
| 75 | 01001010 | 3 | 0.601 | 0.526 | 0.566 |
| 76 | 01001011 | 4 | 0.785 | 0.836 | 0.907 |
| 77 | 01001100 | 3 | 0.545 | 0.525 | 0.53 |
| 78 | 01001101 | 4 | 0.693 | 0.842 | 0.9 |
| 79 | 01001110 | 4 | 0.642 | 0.616 | 0.601 |
| 80 | 01001111 | 5 | 0.827 | 0.912 | 0.939 |
| 81 | 01010000 | 2 | 0.52 | 0.507 | 0.506 |
| 82 | 01010001 | 3 | 0.591 | 0.751 | 0.852 |
| 83 | 01010010 | 3 | 0.563 | 0.522 | 0.531 |
| 84 | 01010011 | 4 | 0.629 | 0.844 | 0.884 |
| 85 | 01010100 | 3 | 0.522 | 0.508 | 0.5 |
| 86 | 01010101 | 4 | 0.591 | 0.786 | 0.846 |
| 87 | 01010110 | 4 | 0.624 | 0.519 | 0.528 |
| 88 | 01010111 | 5 | 0.677 | 0.891 | 0.905 |
| 89 | 01011000 | 3 | 0.517 | 0.501 | 0.524 |
| 90 | 01011001 | 4 | 0.592 | 0.799 | 0.88 |
| 91 | 01011010 | 4 | 0.608 | 0.546 | 0.598 |
| 92 | 01011011 | 5 | 0.726 | 0.874 | 0.908 |
| 93 | 01011100 | 4 | 0.575 | 0.539 | 0.559 |
| 94 | 01011101 | 5 | 0.688 | 0.868 | 0.904 |
| 95 | 01011110 | 5 | 0.657 | 0.655 | 0.665 |
| 96 | 01011111 | 6 | 0.825 | 0.92 | 0.94 |
| 97 | 01100000 | 2 | 0.509 | 0.504 | 0.501 |
| 98 | 01100001 | 3 | 0.637 | 0.776 | 0.841 |
| 99 | 01100010 | 3 | 0.586 | 0.543 | 0.545 |
| 100 | 01100011 | 4 | 0.793 | 0.892 | 0.901 |
| 101 | 01100100 | 3 | 0.561 | 0.537 | 0.514 |
| 102 | 01100101 | 4 | 0.688 | 0.882 | 0.869 |
| 103 | 01100110 | 4 | 0.683 | 0.615 | 0.552 |
| 104 | 01100111 | 5 | 0.859 | 0.974 | 0.934 |
| 105 | 01101000 | 3 | 0.561 | 0.514 | 0.503 |
| 106 | 01101001 | 4 | 0.7 | 0.853 | 0.913 |
| 107 | 01101010 | 4 | 0.676 | 0.612 | 0.595 |
| 108 | 01101011 | 5 | 0.912 | 0.93 | 0.945 |
| 109 | 01101100 | 4 | 0.662 | 0.597 | 0.539 |
| 110 | 01101101 | 5 | 0.805 | 0.95 | 0.96 |
| 111 | 01101110 | 5 | 0.773 | 0.76 | 0.661 |
| 112 | 01101111 | 6 | 0.985 | 0.997 | 0.989 |
| 113 | 01110000 | 3 | 0.508 | 0.508 | 0.502 |
| 114 | 01110001 | 4 | 0.603 | 0.825 | 0.897 |
| 115 | 01110010 | 4 | 0.597 | 0.557 | 0.559 |
| 116 | 01110011 | 5 | 0.705 | 0.923 | 0.937 |
| 117 | 01110100 | 4 | 0.561 | 0.536 | 0.515 |
| 118 | 01110101 | 5 | 0.637 | 0.891 | 0.905 |
| 119 | 01110110 | 5 | 0.673 | 0.644 | 0.578 |
| 120 | 01110111 | 6 | 0.795 | 0.978 | 0.967 |
| 121 | 01111000 | 4 | 0.557 | 0.53 | 0.52 |
| 122 | 01111001 | 5 | 0.674 | 0.876 | 0.928 |
| 123 | 01111010 | 5 | 0.679 | 0.65 | 0.649 |
| 124 | 01111011 | 6 | 0.857 | 0.945 | 0.961 |
| 125 | 01111100 | 5 | 0.683 | 0.619 | 0.572 |
| 126 | 01111101 | 6 | 0.807 | 0.951 | 0.963 |
| 127 | 01111110 | 6 | 0.795 | 0.814 | 0.727 |
| 128 | 01111111 | 7 | 0.986 | 0.998 | 0.992 |
| 129 | 10000000 | 1 | 0.518 | 0.5 | 0.507 |
| 130 | 10000001 | 2 | 0.61 | 0.727 | 0.793 |
| 131 | 10000010 | 2 | 0.563 | 0.512 | 0.52 |
| 132 | 10000011 | 3 | 0.678 | 0.828 | 0.864 |
| 133 | 10000100 | 2 | 0.54 | 0.502 | 0.512 |
| 134 | 10000101 | 3 | 0.629 | 0.769 | 0.819 |
| 135 | 10000110 | 3 | 0.576 | 0.508 | 0.515 |
| 136 | 10000111 | 4 | 0.683 | 0.878 | 0.866 |
| 137 | 10001000 | 2 | 0.525 | 0.503 | 0.52 |
| 138 | 10001001 | 3 | 0.641 | 0.795 | 0.858 |
| 139 | 10001010 | 3 | 0.612 | 0.528 | 0.587 |
| 140 | 10001011 | 4 | 0.777 | 0.876 | 0.895 |
| 141 | 10001100 | 3 | 0.558 | 0.529 | 0.554 |
| 142 | 10001101 | 4 | 0.708 | 0.855 | 0.893 |
| 143 | 10001110 | 4 | 0.645 | 0.61 | 0.615 |
| 144 | 10001111 | 5 | 0.827 | 0.913 | 0.942 |
| 145 | 10010000 | 2 | 0.515 | 0.515 | 0.513 |
| 146 | 10010001 | 3 | 0.626 | 0.772 | 0.847 |
| 147 | 10010010 | 3 | 0.575 | 0.519 | 0.542 |
| 148 | 10010011 | 4 | 0.673 | 0.849 | 0.872 |
| 149 | 10010100 | 3 | 0.524 | 0.501 | 0.504 |
| 150 | 10010101 | 4 | 0.666 | 0.813 | 0.842 |
| 151 | 10010110 | 4 | 0.627 | 0.518 | 0.525 |
| 152 | 10010111 | 5 | 0.729 | 0.893 | 0.9 |
| 153 | 10011000 | 3 | 0.521 | 0.512 | 0.558 |
| 154 | 10011001 | 4 | 0.661 | 0.832 | 0.862 |
| 155 | 10011010 | 4 | 0.619 | 0.545 | 0.643 |
| 156 | 10011011 | 5 | 0.787 | 0.876 | 0.909 |
| 157 | 10011100 | 4 | 0.579 | 0.537 | 0.589 |
| 158 | 10011101 | 5 | 0.766 | 0.884 | 0.919 |
| 159 | 10011110 | 5 | 0.691 | 0.654 | 0.689 |
| 160 | 10011111 | 6 | 0.901 | 0.929 | 0.958 |
| 161 | 10100000 | 2 | 0.525 | 0.498 | 0.534 |
| 162 | 10100001 | 3 | 0.62 | 0.795 | 0.832 |
| 163 | 10100010 | 3 | 0.581 | 0.538 | 0.575 |
| 164 | 10100011 | 4 | 0.718 | 0.917 | 0.912 |
| 165 | 10100100 | 3 | 0.55 | 0.525 | 0.532 |
| 166 | 10100101 | 4 | 0.638 | 0.88 | 0.86 |
| 167 | 10100110 | 4 | 0.63 | 0.614 | 0.596 |
| 168 | 10100111 | 5 | 0.757 | 0.977 | 0.936 |
| 169 | 10101000 | 3 | 0.565 | 0.51 | 0.54 |
| 170 | 10101001 | 4 | 0.668 | 0.864 | 0.893 |
| 171 | 10101010 | 4 | 0.65 | 0.613 | 0.646 |
| 172 | 10101011 | 5 | 0.853 | 0.957 | 0.959 |
| 173 | 10101100 | 4 | 0.628 | 0.592 | 0.58 |
| 174 | 10101101 | 5 | 0.778 | 0.945 | 0.949 |
| 175 | 10101110 | 5 | 0.73 | 0.777 | 0.706 |
| 176 | 10101111 | 6 | 0.94 | 1 | 1 |
| 177 | 10110000 | 3 | 0.514 | 0.507 | 0.518 |
| 178 | 10110001 | 4 | 0.617 | 0.838 | 0.872 |
| 179 | 10110010 | 4 | 0.599 | 0.541 | 0.602 |
| 180 | 10110011 | 5 | 0.702 | 0.935 | 0.928 |
| 181 | 10110100 | 4 | 0.577 | 0.534 | 0.526 |
| 182 | 10110101 | 5 | 0.66 | 0.913 | 0.892 |
| 183 | 10110110 | 5 | 0.669 | 0.649 | 0.622 |
| 184 | 10110111 | 6 | 0.796 | 0.98 | 0.96 |
| 185 | 10111000 | 4 | 0.557 | 0.531 | 0.567 |
| 186 | 10111001 | 5 | 0.676 | 0.884 | 0.922 |
| 187 | 10111010 | 5 | 0.657 | 0.65 | 0.688 |
| 188 | 10111011 | 6 | 0.849 | 0.957 | 0.965 |
| 189 | 10111100 | 5 | 0.673 | 0.613 | 0.616 |
| 190 | 10111101 | 6 | 0.825 | 0.961 | 0.962 |
| 191 | 10111110 | 6 | 0.787 | 0.812 | 0.759 |
| 192 | 10111111 | 7 | 0.972 | 1 | 1 |
| 193 | 11000000 | 2 | 0.516 | 0.499 | 0.491 |
| 194 | 11000001 | 3 | 0.606 | 0.803 | 0.821 |
| 195 | 11000010 | 3 | 0.582 | 0.515 | 0.556 |
| 196 | 11000011 | 4 | 0.698 | 0.891 | 0.873 |
| 197 | 11000100 | 3 | 0.522 | 0.502 | 0.512 |
| 198 | 11000101 | 4 | 0.607 | 0.859 | 0.848 |
| 199 | 11000110 | 4 | 0.605 | 0.537 | 0.533 |
| 200 | 11000111 | 5 | 0.692 | 0.93 | 0.908 |
| 201 | 11001000 | 3 | 0.527 | 0.493 | 0.492 |
| 202 | 11001001 | 4 | 0.626 | 0.845 | 0.862 |
| 203 | 11001010 | 4 | 0.624 | 0.566 | 0.593 |
| 204 | 11001011 | 5 | 0.765 | 0.909 | 0.906 |
| 205 | 11001100 | 4 | 0.57 | 0.548 | 0.555 |
| 206 | 11001101 | 5 | 0.675 | 0.916 | 0.909 |
| 207 | 11001110 | 5 | 0.646 | 0.658 | 0.664 |
| 208 | 11001111 | 6 | 0.831 | 0.937 | 0.949 |
| 209 | 11010000 | 3 | 0.528 | 0.518 | 0.51 |
| 210 | 11010001 | 4 | 0.607 | 0.838 | 0.823 |
| 211 | 11010010 | 4 | 0.594 | 0.532 | 0.552 |
| 212 | 11010011 | 5 | 0.673 | 0.896 | 0.866 |
| 213 | 11010100 | 4 | 0.517 | 0.505 | 0.508 |
| 214 | 11010101 | 5 | 0.631 | 0.884 | 0.856 |
| 215 | 11010110 | 5 | 0.632 | 0.561 | 0.563 |
| 216 | 11010111 | 6 | 0.706 | 0.935 | 0.911 |
| 217 | 11011000 | 4 | 0.53 | 0.524 | 0.553 |
| 218 | 11011001 | 5 | 0.615 | 0.858 | 0.844 |
| 219 | 11011010 | 5 | 0.617 | 0.578 | 0.651 |
| 220 | 11011011 | 6 | 0.752 | 0.907 | 0.909 |
| 221 | 11011100 | 5 | 0.59 | 0.58 | 0.612 |
| 222 | 11011101 | 6 | 0.709 | 0.923 | 0.908 |
| 223 | 11011110 | 6 | 0.684 | 0.709 | 0.733 |
| 224 | 11011111 | 7 | 0.857 | 0.948 | 0.959 |
| 225 | 11100000 | 3 | 0.533 | 0.49 | 0.51 |
| 226 | 11100001 | 4 | 0.639 | 0.866 | 0.873 |
| 227 | 11100010 | 4 | 0.606 | 0.559 | 0.573 |
| 228 | 11100011 | 5 | 0.772 | 0.957 | 0.919 |
| 229 | 11100100 | 4 | 0.574 | 0.539 | 0.517 |
| 230 | 11100101 | 5 | 0.662 | 0.922 | 0.898 |
| 231 | 11100110 | 5 | 0.698 | 0.661 | 0.6 |
| 232 | 11100111 | 6 | 0.843 | 0.99 | 0.953 |
| 233 | 11101000 | 4 | 0.591 | 0.504 | 0.525 |
| 234 | 11101001 | 5 | 0.694 | 0.901 | 0.918 |
| 235 | 11101010 | 5 | 0.696 | 0.643 | 0.637 |
| 236 | 11101011 | 6 | 0.907 | 0.96 | 0.96 |
| 237 | 11101100 | 5 | 0.668 | 0.612 | 0.573 |
| 238 | 11101101 | 6 | 0.805 | 0.967 | 0.964 |
| 239 | 11101110 | 6 | 0.804 | 0.793 | 0.734 |
| 240 | 11101111 | 7 | 0.986 | 1 | 1 |
| 241 | 11110000 | 4 | 0.527 | 0.501 | 0.501 |
| 242 | 11110001 | 5 | 0.618 | 0.884 | 0.877 |
| 243 | 11110010 | 5 | 0.628 | 0.588 | 0.61 |
| 244 | 11110011 | 6 | 0.734 | 0.951 | 0.929 |
| 245 | 11110100 | 5 | 0.576 | 0.53 | 0.522 |
| 246 | 11110101 | 6 | 0.67 | 0.941 | 0.91 |
| 247 | 11110110 | 6 | 0.704 | 0.687 | 0.646 |
| 248 | 11110111 | 7 | 0.834 | 0.986 | 0.972 |
| 249 | 11111000 | 5 | 0.593 | 0.548 | 0.58 |
| 250 | 11111001 | 6 | 0.7 | 0.91 | 0.919 |
| 251 | 11111010 | 6 | 0.699 | 0.694 | 0.696 |
| 252 | 11111011 | 7 | 0.891 | 0.965 | 0.962 |
| 253 | 11111100 | 6 | 0.719 | 0.66 | 0.651 |
| 254 | 11111101 | 7 | 0.836 | 0.973 | 0.968 |
| 255 | 11111110 | 7 | 0.821 | 0.832 | 0.791 |
| 256 | 11111111 | 8 | 0.999 | 1 | 1 |

**Supplementary Table 1**. Classification accuracy across all 256 fragmented stimulus groups. Each row corresponds to one of the 256 unique fragmented stimulus configurations used in the experiments, defined by different combinations of contour fragments. Columns report the binary code and fragment availability for each group and classification accuracy for Alexnet, Inception, and Resnet. Rows are ordered consistently across networks.

| **Parameter** | **Value** |
| --- | --- |
| Optimizer | Adam |
| Loss Function | Categorical Cross-Entropy |
| Initial Learning Rate | 0.0001 |
| Mini-Batch Size | 64 |
| Shuffle | Every Epoch |
| Max Epochs | 4 (Inception)  5 (Resnet)  10 (Alexnet) |
| Gradient Threshold Method | 0.0001 L2 Norm |
| Output Network | Best Validation |

**Supplementary Table 2.** Training hyperparameters and optimization settings used for the network training. These parameters were used consistently across models unless otherwise specified.
